# Cyberspace Supplementary Health Services and Patient Loyalty: The Role of Patient Experience in Saudi Arabia

**DOI:** 10.3390/healthcare14162512

**Published:** 2026-08-12

**Authors:** Alaeddin Ahmad, Nizar Alsubahi, Fahad Alhazmi, Amani Al-Refai, Sara Fuad Talafha, Mohannad Alkhateeb, Mahmoud Alfatafta

**Affiliations:** 1 Department of Marketing, School of Business, The University of Jordan, Amman 11942, Jordan; 2 Department of Health Service and Hospital Administration, Faculty of Economics and Administration, King Abdul Aziz University, Jeddah 21589, Saudi Arabia; 3 Department of Health Services Research, Care and Public Health Research Institute—CAPHRI, Maastricht University Medical Center, Faculty of Health, Medicine and Life Sciences, Maastricht University, P.O. Box 616, 6200 MD Maastricht, The Netherlands; 4 Department of Basic Sciences and Humanities, Faculty of Arts and Humanities, Applied Science Private University, Amman 11937, Jordan; 5 Faculty of Pharmacy Al-Ahliyya Amman University, Amman 19328, Jordan; 6 School of Public Health and Community Medicine, Institute of Medicine, University of Gothenburg, 40530 Gothenburg, Sweden; 7 Department of Prosthetics and Orthotics, School of Rehabilitation Sciences, The University of Jordan, Amman 11942, Jordan

**Keywords:** cyberspace supplementary health services, E-Information, E-Order Taking, E-Consultation, E-Billing, E-Payment, patient experience, patient loyalty, private hospitals, Jeddah, Saudi Arabia

## Abstract

**Background:** Digital transformation has changed how patients interact with hospitals, making online supplementary services an important part of perceived service quality and relationship outcomes. This study examined the association between cyberspace supplementary health services (CSS), conceptualized as the Online Flower of Service (OFOS), and patient loyalty in private hospitals in Jeddah, Saudi Arabia, and evaluated whether patient experience mediates this relationship. **Methods:** A cross-sectional survey was conducted among adult inpatients and outpatients who had used at least one online hospital service. Data were collected electronically between 10 February 2026 and 10 May 2026 using a structured Arabic questionnaire administered via Google Forms. The final sample included 730 complete responses. CSS was measured across five digital supplementary dimensions (E-Information, E-Order Taking, E-Consultation, E-Billing, and E-Payment), alongside patient experience and patient loyalty, using five-point Likert scales. The measurement model was evaluated using confirmatory factor analysis, and the structural relationships were tested using structural equation modeling. **Results:** CSS demonstrated a significant positive association with patient loyalty (β = 0.282, *p* < 0.001) and a strong positive association with patient experience (β = 0.591, *p* < 0.001). Patient experience was also positively associated with patient loyalty (β = 0.431, *p* < 0.001), supporting its mediating role in the CSS–patient loyalty relationship. **Conclusions:** Digitally delivered CSS components were positively associated with patient loyalty, with patient experience serving as an important mediating mechanism. Strengthening online information access, appointment-related processes, digital consultation, billing transparency, and payment convenience may be associated with more favorable patient experiences and stronger loyalty intentions in private hospital settings in Saudi Arabia.

## 1. Introduction

Digital transformation has fundamentally reshaped healthcare systems worldwide [1]. The rapid integration of electronic health records, online appointment systems, digital billing, teleconsultations, and electronic payment platforms has altered how patients interact with healthcare providers and evaluate service quality [1,2,3]. Recent systematic reviews confirm that digital health infrastructure is increasingly associated with improved patient satisfaction, accessibility, and perceived quality of care [3,4,5]. As healthcare delivery becomes more digitally embedded, patients no longer assess services solely on clinical outcomes but also on the efficiency, transparency, and usability of digital service processes.

Within Saudi Arabia, digital healthcare expansion has accelerated under the Vision 2030 Health Sector Transformation Program, which prioritizes digital health technologies, patient-centered care, and improved access to healthcare services [6,7,8,9]. Recent evidence demonstrates substantial growth in the adoption of digital health platforms, electronic health records, telemedicine services, online appointment systems, and virtual hospitals across the Kingdom [10,11,12,13]. Initiatives such as Seha Virtual Hospital and other national digital health programs have transformed how healthcare services are delivered and accessed, increasing patient engagement with electronic healthcare platforms [12]. As a result, patients are increasingly interacting with healthcare providers through digital channels before, during, and after clinical encounters. Despite this rapid transformation, evidence remains limited regarding how different digital service components collectively influence patient experience and loyalty within Saudi private hospitals, particularly in Jeddah, where the private healthcare sector plays a major role in service delivery.

The supplementary services (SS) model, originally proposed by Lovelock, conceptualizes supplementary services that support core service delivery [14]. Traditionally, these supplementary services include information, consultation, order taking, billing, and payment. As healthcare services become increasingly digitalized, these service elements are now frequently delivered through electronic platforms. In the present study, these digitally delivered supplementary services are conceptualized as Cyberspace Supplementary Services (CSS), comprising E-Information, E-Order Taking, E-Consultation, E-Billing, and E-Payment. While service quality theory has extensively examined the influence of reliability, responsiveness, assurance, empathy, and tangibility on healthcare outcomes [15], considerably less attention has been given to the collective role of digital supplementary services in shaping patient experience and loyalty.

Emerging evidence suggests that digital service quality positively influences patient perceptions, engagement, and behavioral intentions [16,17,18]. Electronic appointment systems, teleconsultation services, digital billing platforms, and online payment systems have been associated with improved convenience, reduced waiting times, enhanced accessibility, and more efficient healthcare delivery [17,19,20,21]. However, most previous studies have focused on individual digital technologies rather than examining digital supplementary services as an integrated service ecosystem. Consequently, limited evidence exists regarding how multiple digital service dimensions jointly influence patient loyalty within healthcare settings.

Patient experience has been identified as a central mediator in healthcare quality assessment frameworks [22,23]. Patient experience encompasses patients’ perceptions across their entire care journey, including communication clarity, service coordination, accessibility, and engagement [23]. Strong empirical evidence confirms that positive patient experiences are associated with higher satisfaction, stronger trust, continued service use, and greater loyalty intentions [24]. Despite this evidence, limited research has examined whether patient experience serves as a mechanism through which cyberspace supplementary services influence patient loyalty in digitally enabled healthcare environments.

Accordingly, this study examines the association between Cyberspace Supplementary Services (CSS) and patient loyalty among adults attending private hospitals in Jeddah, Saudi Arabia, while evaluating the mediating role of patient experience. In this study, CSS is conceptualized as a digitally adapted form of the Flower of Service framework and comprises E-Information, E-Order Taking, E-Consultation, E-Billing, and E-Payment. Patient loyalty is defined as patients’ intention to continue using the hospital’s services, revisit the hospital when future healthcare needs arise, and maintain an ongoing relationship with the healthcare provider.

By integrating the Flower of Service framework with contemporary digital healthcare research, this study contributes to the literature by examining digital supplementary services as an integrated service system rather than as isolated technologies. It further investigates patient experience as an explanatory mechanism linking CSS to patient loyalty. The study therefore addresses an important gap in understanding how digitally delivered supplementary services are associated with patients’ future behavioral intentions within the Saudi private healthcare context.

## 2. Methods

### 2.1. Design

This study employed a cross-sectional quantitative design to examine the relationships between Cyberspace Supplementary Services (CSS), patient experience, and patient loyalty among adults attending private hospitals in Jeddah, Kingdom of Saudi Arabia. The study was grounded in the Flower of Service framework proposed by Lovelock, which emphasizes the role of supplementary services in supporting core service delivery and shaping customer outcomes [14]. In the context of digital healthcare, the framework was adapted to reflect electronically delivered supplementary services, conceptualized as Cyberspace Supplementary Services (CSS).

The proposed conceptual model included CSS as the independent variable, patient loyalty as the dependent variable, and patient experience as a mediating variable. CSS was operationalized through five digital service dimensions: E-Information, E-Order Taking, E-Consultation, E-Billing, and E-Payment. Based on service quality theory and emerging evidence on digital healthcare services, it was hypothesized that CSS would positively influence patient loyalty both directly and indirectly through patient experience.

A survey-based approach was adopted to collect primary data from patients who had previously used digital services provided by private hospitals. This design was considered appropriate because it enables the assessment of perceptions, experiences, and behavioral outcomes within real-world healthcare settings and has been widely used in healthcare service quality and patient experience research [22,23].

### 2.2. Participants

The study was conducted among adult patients attending private hospitals in Jeddah, Kingdom of Saudi Arabia. Both inpatient and outpatient populations were included because the study focused on experiences with digital supplementary healthcare services that are accessible across the patient journey, regardless of admission status. These services included electronic appointment scheduling, online consultation services, electronic billing systems, electronic payment platforms, and online health information portals.

Inclusion criteria required participants to be aged 18 years or older, to have visited a private hospital in Jeddah during the data collection period, and to have prior experience with at least one online hospital service. Individuals who had not used any digital hospital services or who were unable to complete the questionnaire independently were excluded from the study.

A non-probability convenience sampling technique was employed because no comprehensive sampling frame was available for private hospital patients who had prior experience with digital healthcare services. Participants were recruited through collaborating private hospitals in Jeddah. Hospital administrative and patient-relations departments assisted in distributing the survey link electronically to eligible patients through hospital communication channels. In addition, QR codes linked to the survey were made available within participating hospitals and their digital communication platforms. Participation was entirely voluntary, and no incentives were provided. Because the questionnaire was disseminated through multiple electronic distribution channels, the total number of individuals who received the survey invitation could not be determined; therefore, a response rate could not be calculated. As a result of the convenience sampling strategy, the study sample may not be fully representative of all patients attending private hospitals in Jeddah, and the findings should be interpreted accordingly.

### 2.3. Data Collection

Data collection was conducted between 10 February 2026 and 10 May 2026. During this period, 730 fully completed questionnaires meeting the eligibility criteria were received and included in the final analysis. This sample size exceeded the minimum recommended sample size for structural equation modelling and provided adequate statistical power for testing the proposed conceptual model. Demographic information collected included age, gender, educational level, and self-reported level of technology knowledge.

The study adhered to STROBE guidelines for observational studies (EQUATOR Network). The questionnaire was administered in Arabic, the primary language of the target population. Where original items were adapted from English-language sources, a forward–backward translation procedure was conducted by bilingual experts to ensure linguistic equivalence and conceptual consistency.

All responses were collected anonymously. No personally identifiable information was obtained. Participation was voluntary, and electronic informed consent was obtained from all participants prior to survey completion.

Ethical approval for this study was granted by the Research Ethics Committee (REC), Faculty of Medicine, King Abdulaziz University Hospital, Jeddah, Kingdom of Saudi Arabia (Reference No. 97-26; NCBE Registration No. HA-02-J-008). The study was approved as a non-intervention cross-sectional study and was conducted in accordance with the regulations of the National Committee of Bioethics (NCBE) and Good Clinical Practice (GCP) guidelines.

### 2.4. Measures

All constructs were measured using a five-point Likert scale ranging from 1 (strongly disagree) to 5 (strongly agree). The CSS construct comprised five dimensions: E-Information, E-Order Taking, E-Consultation, E-Billing, and E-Payment. Patient experience was measured using five items reflecting patients’ perceptions of their overall care journey. Patient loyalty was measured using five items assessing overall loyalty with healthcare supplementary services.

Measurement items were adapted from the literature and previously validated instruments in the supplementary services in health service and digital health services, patient experience, and patient loyalty [17,18,19,20,21,23,25,26,27].

Content validity was evaluated through expert review by three academic specialists in health services administration and service management, who evaluated the questionnaire for relevance, clarity, wording, cultural appropriateness, and consistency with the study objectives. Based on their recommendations, several items were linguistically refined to improve clarity and readability, redundant wording was removed, and minor contextual adaptations were made to better reflect the Saudi healthcare setting. These revisions enhanced the comprehensibility of the instrument while preserving the underlying constructs and conceptual framework.

The questionnaire was administered in Arabic. Where items were originally developed in English, a forward–backward translation procedure was performed by bilingual experts to ensure linguistic equivalence and conceptual consistency. The translated version was reviewed and compared with the original instrument to resolve discrepancies and ensure cultural appropriateness.

Following expert review and linguistic validation, the questionnaire was pilot tested with 15 participants representative of the target study population to evaluate the clarity, readability, comprehensibility, questionnaire flow, completion time, and overall feasibility of the instrument prior to the main study. Participants were also invited to provide comments on item wording, response options, and the overall layout of the questionnaire. Based on the feedback received, minor revisions were made to improve wording, grammar, readability, and formatting, while preserving the conceptual meaning of the questionnaire items. No items were added or removed, and the underlying construct structure remained unchanged. A summary of the pilot participant feedback and the revisions implemented is presented in File S1. Data collected during the pilot phase were not included in the final analysis.

Following the validation process, the final questionnaire consisted of 25 items distributed across the study constructs. The complete survey instrument is provided in the Supplementary Materials (File S2).

### 2.5. Data Analysis

Statistical analyses were performed using IBM SPSS Statistics version 28 and IBM AMOS version 26. Descriptive statistics were used to summarize the demographic characteristics of the participants and the study variables (Table 1). The internal consistency of the study constructs was evaluated using Cronbach’s alpha coefficient.

Prior to conducting the main analyses, data were screened for completeness, outliers, and normality. Normality was assessed using skewness and kurtosis statistics. Values within the recommended ranges were considered indicative of acceptable univariate normality for structural equation modelling.

The measurement model was evaluated using Confirmatory Factor Analysis (CFA). Convergent validity was examined by calculating the Average Variance Extracted (AVE) and Composite Reliability (CR), with threshold values of ≥0.50 for AVE and ≥0.70 for CR, indicating acceptable convergent validity [28]. Discriminant validity was evaluated by examining inter-construct correlations and ensuring that correlation coefficients did not exceed 0.85.

Structural equation modeling (SEM) was then used to examine the proposed relationships among Cyberspace Supplementary Services (CSS), Patient Experience (PE), and Patient Loyalty (PL), including the mediating effect of patient experience. Model adequacy was assessed using several goodness-of-fit indices, namely the Comparative Fit Index (CFI), Tucker–Lewis Index (TLI), Root Mean Square Error of Approximation (RMSEA), and the chi-square to degrees of freedom ratio (χ^2^/df). A satisfactory model fit was indicated by CFI and TLI values of ≥0.90, an RMSEA value of ≤0.08, and a χ^2^/df ratio of ≤5 [28]. Statistical significance was determined at *p* < 0.05.

Because all study variables were collected using a single self-administered questionnaire, Harman’s single-factor test was performed to assess the potential influence of common method variance. Following the recommendation of Podsakoff et al. 2003 [29], common method bias was considered unlikely if the first unrotated factor explained less than 50% of the total variance.

## 3. Results

### 3.1. Demographic

The age, gender, educational level, and knowledge in technology were used as demographic factors to analyze the profile of respondents by the number of respondents and percent of respondents based on the demographic factors.

Table 1 describes the profile of respondents, based on gender, educational level, age, and knowledge of technology.

According to the results in Table 1, the percent of respondents from males was 34.8% and 65.2% from females, regarding the educational level the majority was the holders of bachelor’s degree with percent of 60.8% of the total respondents, 20.8% have high degrees, and 18.4% are with the category of diploma and less. According to the age distribution, the majority of respondents was the age category of 51 years and more with percent of 34.2%, and the lowest was the age category of 20 years and less with ratio 3.3%. 40.8% of respondents have high knowledge in technology, 54.0% have moderate knowledge in technology, and 5.2% have low knowledge in technology.

### 3.2. Confirmatory Factor Analysis (CFA) for Validity and Reliability

Table 2 presents the descriptive statistics, including the mean and standard deviation, for the independent variables representing Cyberspace Supplementary Services (CSS), the mediator variable (Patient Experience; PE), and the dependent variable (Patient Loyalty; PL). Mean scores ranged from 3.86 to 4.09. The standardized factor loadings obtained from the confirmatory factor analysis ranged from 0.781 to 0.850, all exceeding the recommended minimum value of 0.50 [28]. Convergent validity was further supported by Composite Reliability (CR) values between 0.839 and 0.869 and Average Variance Extracted (AVE) values between 0.521 and 0.583, both of which exceeded the recommended thresholds of 0.70 and 0.50, respectively [29]. These findings indicate satisfactory internal consistency and adequate convergent validity for all latent constructs.

To assess the potential influence of common method variance, Harman’s single-factor test was conducted using all questionnaire items. The unrotated exploratory factor analysis showed that the first factor accounted for 33.4% of the total variance, which is below the recommended threshold of 50%. Therefore, common method variance was unlikely to have materially influenced the study findings, Podsakoff et al., 2003 [29].

The overall structural model demonstrated an acceptable fit to the data. The chi-square statistic was χ^2^ = 538.124, with a χ^2^/df ratio of 2.564, indicating a good model fit. Additional goodness-of-fit indices also supported the adequacy of the model, including CFI = 0.924, TLI = 0.910, NFI = 0.902, IFI = 0.905, and RMSEA = 0.075. Collectively, these indices indicate that the proposed measurement and structural models provided an acceptable representation of the observed data.

Furthermore, Cronbach’s alpha coefficients ranged from 0.822 to 0.859, exceeding the recommended threshold of 0.70 and indicating satisfactory internal consistency for all constructs. Discriminant validity was evaluated using the Average Variance Extracted (AVE), with the results presented in Table 2. All AVE values exceeded the recommended threshold of 0.50 and were greater than the corresponding inter-construct correlations, confirming that each construct was empirically distinct from the others.

### 3.3. Discriminant Validity Assessment

Discriminant validity was assessed using three complementary approaches: the construct correlation matrix, the Fornell–Larcker criterion, and the Heterotrait–Monotrait (HTMT) ratio. All inter-construct correlations were below the recommended threshold of 0.85. Furthermore, the square root of the AVE for each construct exceeded its correlations with all other constructs, thereby satisfying the Fornell–Larcker criterion. Finally, all HTMT values were below the recommended cutoff value of 0.85, with the highest HTMT value observed between Patient Experience and Patient Loyalty (HTMT = 0.825). Collectively, these findings provide strong evidence that the constructs exhibit adequate discriminant validity despite the conceptual relatedness of Patient Experience and Patient Loyalty. Detailed results are presented in File S3 (Tables S6–S8).

### 3.4. Path Analysis of (SEM)

Following confirmation of the measurement model through Confirmatory Factor Analysis (CFA), Structural Equation Modeling (SEM) was performed to evaluate the proposed research model and test the study hypotheses. The hypotheses examined were as follows:

**H1.**  *There is a significant positive impact of CSS on PL.*

**H1a.**  *There is a significant positive impact of E-Information on PL.*

**H1b.**  *There is a significant positive impact of E-Order taking (E-Appointment) on PL.*

**H1c.**  *There is a significant positive impact of E-Consultation on PL.*

**H1d.**  *There is a significant positive impact of E-Billing on PL.*

**H1e.**  *There is a significant positive impact of E-Payment on PL.*

**H2.**  *There is a significant positive impact of CSS on PE.*

**H3.**  *There is a significant positive impact of PE on PL.*

**H4.**  *There is a significant positive impact of CSS on PL mediating by PE.*

Main Hypotheses (H1, H2, H3)

**Figure 1:**
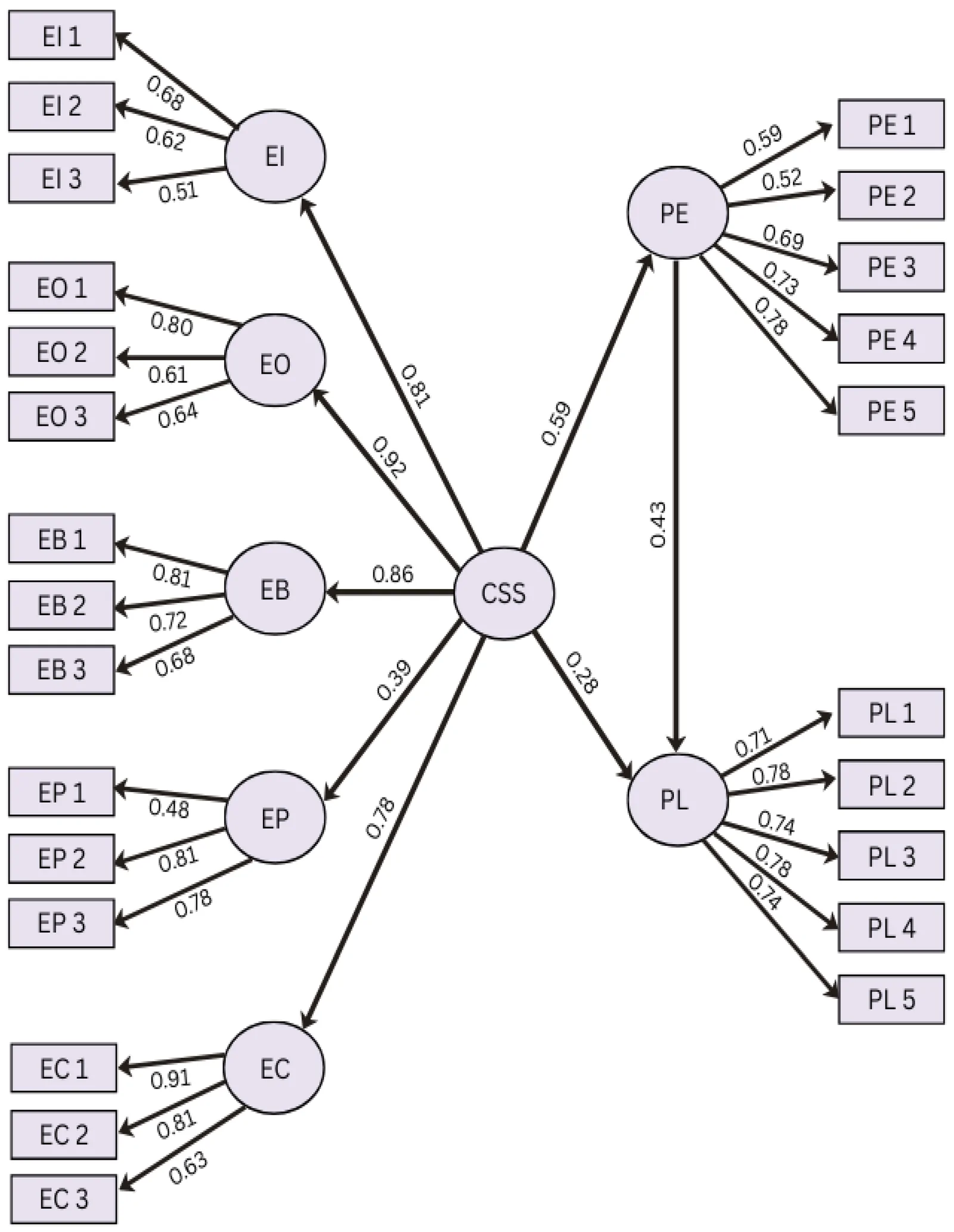
Output of SEM. CSS = Cyberspace supplementary health services, EI = E-Information, EO = E-Order Taking, EC = E-Consultation, EB = E-Billing, and EP = E-Payment, PL = Patient loyalty, PE = Patient experience.

Structural equation modeling (SEM) was performed to examine the proposed relationships among Cyberspace Supplementary Services (CSS), Patient Experience (PE), and Patient Loyalty (PL), including the mediating role of patient experience. Figure 1 illustrates the final structural model, and Table 3 presents the standardized regression coefficients (β) and associated statistical results for each hypothesized path.

Table 3 shows that CSS was significantly associated with PL (*p* < 0.001, Beta = 0.282). Similarly, CSS was significantly associated with PE (*p* < 0.001, Beta = 0.591). In the same way, PE was significantly associated with PL (*p* < 0.001, Beta = 0.431). The value of R^2^ of PL was 0.462, which means that 46.2% of PL was explained by CSS dimensions (Table 3).

### 3.5. Direct Effect Sub-Hypotheses (H1a, H1b, H1c, H1d, H1e)

Based on the output of path analysis as described in Figure 1.

- EI has a significant positive effect on PL, (β = 0.181, *p* < 0.01).
- EO taking has a significant positive effect on PL, (β = 0.921, *p* < 0.05).
- EB has a significant positive effect on PL, (β = 0.866, *p* < 0.01).
- EP has a significant positive effect on PL, (β = 0.391, *p* < 0.05).
- EC has a significant positive effect on PL, (β = 0.781, *p* < 0.05).

## 4. Discussion

This study demonstrates a significant relationship between CSS, patient experience, and patient loyalty in healthcare settings. It is important to note that digital service elements play a crucial role in shaping patients’ perceptions of healthcare quality. This observation is supported by both foundational research on supplementary services and recent systematic reviews examining the digital transformation of healthcare, which are positively correlated with patient experience, satisfaction, and loyalty [18,19,21,27,30,31].

The results support Hypothesis H1, as CSS exerted a statistically significant positive effect on patient loyalty (*p* < 0.001, β = 0.282). Our findings suggest that patients are increasingly considering not just clinical outcomes, but also the efficiency and usability of online service processes when evaluating healthcare. This shift in patient priorities may reflect broader changes in expectations driven by digitization, as demonstrated by recent studies showing significant improvements in patient loyalty, particularly regarding accessibility and service quality, in Saudi Arabia and beyond [13,32]. The findings further suggest that the key CSS dimensions collectively were associated with higher patient loyalty by improving service accessibility, transparency, and operational convenience. These enhancements not only promote more positive patient perceptions but can also lead to improved patient outcomes, such as greater adherence to treatment plans, higher follow-up rates, and reduced administrative errors, supporting the transition from traditional service quality models toward digitally embedded service delivery, reinforcing and extending the fundamental work of Parasuraman et al. (1985) [15] into contemporary digital healthcare contexts. Also, consistent literature on how digital service transformation enhances patient-centric care and satisfaction outcomes [16]. To better understand how CSS affects patient loyalty, the study looked at each CSS dimension separately. Hypothesis H1a was supported, showing that E-Information was significantly positively associated with patient loyalty (β = 0.181, *p* < 0.01). This means that having accurate, timely, and easy-to-access health information helps patients feel more in control and satisfied. This finding matches earlier studies on the importance of digital information quality in healthcare [27,33]. Hypothesis H1b was also supported, as E-Order Taking (E-Appointment) had a significant positive effect on patient loyalty (β = 0.921, *p* < 0.05). This shows that easier appointment scheduling and shorter wait times through digital platforms make patients feel services are more effective and convenient. This finding matches previous studies on the importance of E-appointment on stakeholders’ experiences, perceptions and satisfaction in healthcare. Other studies by Munyaka et al. (2025) [21] and Zhou et al. (2025) [16] found that integrating digital payment and billing systems enhances the patient experience by streamlining administrative processes and reducing wait times. Correspondingly, Sagare et al. (2023) [34] found similar results.

Hypothesis H1c was supported, as E-Consultation exhibits a statistically significant effect on patient loyalty (β = 0.781, *p* < 0.05). This finding consistent with previous studies demonstrating positive outcomes for telemedicine and online consultation services [16,18,31]. For example, recent research has highlighted that local healthcare cultures that prioritize in-person interactions may contribute to patient reluctance toward remote consultations [20]. Additional factors, such as familiarity with digital health technologies, socioeconomic status, literacy levels, and trust in digital platforms, also shape patient acceptance and perceptions of E-Consultation [35].

Hypothesis H1d was supported, as E-Billing had a significant positive effect on patient loyalty (β = 0.866, *p* < 0.01). This finding illustrates the importance of transparent, integrated billing systems in lowering administrative complexity and improving the overall service experience. Prior research supports this view, demonstrating that effective electronic billing systems strengthen operational efficiency and reduce patient frustration with financial procedures [17]. Similarly, Hypothesis H1e was supported, with E-Payment showing a statistically significant, though comparatively weaker, positive association with patient loyalty (β = 0.391, *p* < 0.05). Indicating that while digital payment options contribute to convenience and transaction effectiveness, they may be perceived as basic service expectations rather than strong differentiators of loyalty. This interpretation corresponds to the findings of Munyaka et al. (2025) [21]; and Zhou et al. (2025) [16].

Although all CSS dimensions demonstrated statistically significant positive associations with patient loyalty, the magnitude of these associations differed considerably. E-Order Taking, E-Billing, and E-Consultation showed substantially stronger standardized effects than E-Information and E-Payment, suggesting that patients may place greater value on digital services that directly facilitate access to care and simplify administrative processes than on information-related functions alone. These findings indicate that not all digital supplementary services contribute equally to patient loyalty, highlighting the importance of prioritizing digital functions that directly influence patients’ interactions with healthcare providers. Nevertheless, these findings should be interpreted with caution because the relative strength of the associations may also reflect overlap between closely related aspects of digitally delivered healthcare services and patient evaluations.

Regarding Hypothesis H2, which proposes that CSS has a significant positive impact on patient experience, the hypothesis was supported, as CSS exhibited a strong, statistically significant positive effect on patient experience (β = 0.591, *p* < 0.001). This finding indicates that digitally delivered service elements substantially shape how patients experience and engage with healthcare services across different stages of their care pathway.

The findings are consistent with Zhou et al. (2025) [16], who reported that service digitalization improves patient satisfaction. Extending this perspective to a digital healthcare context, the present study demonstrates that online service features, such as information provision, appointment management, billing, and payment systems, jointly enhance the overall patient experience.

Hypothesis H3 was also supported, as patient experience demonstrated a statistically significant positive association with patient loyalty (β = 0.431, *p* < 0.001). This result indicates that patients’ overall experiences play a central role in shaping their loyalty with healthcare services, extending beyond isolated service encounters to encompass the entire care journey. In accordance with established healthcare literature that accentuates the importance of patient experience in evaluating care quality and patient loyalty [26,36]. Prior studies by Gleeson et al. (2016) [24] demonstrated that patient perceptions of the care process significantly influence overall satisfaction and loyalty, while Al-Jabri et al. (2021) [37] showed that patients’ perceptions of their experiences strongly influence their judgments of the quality of care received. These findings reinforce the centrality of patient-oriented approaches in enhancing satisfaction and health-related outcomes. Significantly, the present study expands this body of research by confirming the mediating role of patient experience within a digital healthcare context. The results suggest that CSS is associated with patient loyalty primarily through patient experience rather than through direct effects alone. The finding underscores the importance of managing both clinical and digital channels to ensure a coherent, positive patient experience. In this sense, the results highlight that delivering satisfactory patient experiences at every stage of the healthcare pathway, including online interactions, may strengthen patient loyalty and support longer-term relationships with healthcare providers in digitally enabled healthcare environments.

The structural model explained a substantial proportion (46.2%) of the variance in patient loyalty, indicating that cyberspace supplementary services and patient experience are important determinants of patient loyalty in private healthcare settings. However, patient loyalty is a multifaceted construct, and the remaining unexplained variance suggests that additional organizational, service-related, and individual factors, such as trust, perceived service quality, healthcare provider reputation, and patient characteristics, may also influence loyalty. Future research should incorporate these factors to develop more comprehensive explanatory models.

### 4.1. Practical Implication

The findings of this study offer several practical implications for decision makers and leaders in the healthcare industry. To enhance patient experience and loyalty, healthcare organizations are advised to prioritize investing in digital infrastructure, including integrated electronic health record systems that enable patients to view their medical information, schedule appointments, and communicate with healthcare providers online. Additionally, investment in online service platforms with user-friendly, accessible interfaces is recommended. Given that e-Order Taking, e-Billing, and e-Consultation demonstrated stronger associations with patient loyalty than e-Information and e-Payment, healthcare organizations should prioritize the development and optimization of these high-impact digital services, as they are likely to yield greater improvements in patient loyalty and overall patient experience.

Improving the quality of digital systems can facilitate easier access to health information, streamline online processes, and support a smooth transition from traditional service delivery channels to digital platforms. In addition, healthcare strategies should be designed with a strong patient-centered focus, emphasizing patients’ needs, preferences, and expectations. To support continuous improvement in healthcare service quality, healthcare organizations should encourage effective communication among patients and both administrative and clinical staff, and actively incorporate patient feedback into service design and evaluation. Establishing structured feedback protocols can help healthcare providers identify service gaps and refine digital processes to better meet patient expectations.

### 4.2. Study Limitations

Despite the contributions of this study, some limitations should be acknowledged. Firstly, the research was conducted in private hospitals in Jeddah, Saudi Arabia, using a convenience sample of patients who had previously used at least one digital hospital service. In addition, the study sample was predominantly composed of participants with a bachelor’s degree or higher and moderate to high levels of technology knowledge. Consequently, the findings may not be fully generalizable to public healthcare institutions, other healthcare settings, or patients with lower educational attainment or limited experience using digital healthcare services. Secondly, the adoption of a cross-sectional research design limits the ability to make causal inferences; therefore, the relationships identified in this study should be interpreted as associations rather than definitive cause-and-effect links. Additionally, this temporal limitation means that the study captures patient perceptions at only one point in time, which may limit understanding of how these perceptions evolve as digital healthcare services develop. Thirdly, the study focused on a limited set of CSS dimensions due to the relative scarcity of prior empirical research in this area. While this restriction adds to the research’s novelty, it may yield an incomplete picture by excluding other potentially relevant digital service elements, thereby limiting the comprehensiveness of the findings. Finally, reliance on self-reported survey data may introduce response bias, as patient perceptions and evaluations are subjective and may not fully represent objective service quality. Future studies could extend the current research in several directions. Primarily, additional performance outcomes, such as patient trust, and perceived value, could be examined as dependent variables to yield a more comprehensive understanding of the outcomes associated with CSS in the healthcare context. Moreover, the proposed research model could be applied to other service sectors, including banking, insurance, and telecommunications, to assess the generalizability of the findings beyond the healthcare context and to compare the roles of digital service quality across industries. Additionally, the findings were based on analyses conducted at the aggregate level and did not examine potential differences across demographic subgroups, such as age, educational attainment, or level of digital literacy. Although the overall model provides valuable evidence regarding the relationships among the study variables, these relationships may vary across different patient populations. Future research should employ multi-group structural equation modeling or moderation analyses to investigate whether demographic characteristics influence the strength of these relationships.

## 5. Conclusions

This study offers empirical evidence that CSS is strongly associated with shaping patient loyalty by being associated with patient experience in healthcare settings. The data confirm that patients’ evaluations of healthcare services are increasingly formed not only by clinical outcomes, but also by the quality of digitally delivered service processes across the patient journey. The findings point to the key role of patient experience as an important mechanism linking CSS to patient loyalty. Precisely, CSS dimensions were found to meaningfully contribute to patient loyalty, stressing the importance of accessible information, operational efficiency, and transparency in digitally enabled healthcare environments. The study reveals the growing importance of patient-focused and digitally integrated service delivery in contemporary healthcare systems. By advancing online service processes and aligning them with patient expectations, healthcare organizations can improve patient experiences and loyalty. These outcomes aid a deeper understanding of how digital service quality is associated with patient perceptions and provide a cornerstone for future research and practice in digital healthcare service management. Future research could investigate alternative model specifications by examining the moderating role of patient experience in the relationship between CSS and patient loyalty.

## Figures and Tables

**Table 1:** Demographic analysis of respondents (n = 730).

Item	Category	Frequency	Percent
Gender	Female	476	65.2
	Male	254	34.8
	Total	730	100.0
Educational level	Bachelor’s degree	444	60.8
	High degrees	152	20.8
	Diploma or less	134	18.4
	Total	730	100
Age (years)	20 or less	24	3.3
	21–30	154	21.1
	31–40	152	20.8
	41–50	150	20.5
	51 and more	250	34.2
	Total	730	100.0
Knowledge of Technology	High knowledge in technology	298	40.8
	Medium knowledge in technology	394	54.0
	Low knowledge in technology	38	5.2
	Total		100.0

**Table 2:** Measurement Model Reliability and Validity Results.

Latent Variable	Indicator	FL	FLS	AVE (>0.50)	CR (>0.70)	Cronbach’s Alpha
CSS Mean = 3.86; SD = 0.728 Discriminant Validity Assessment = 0.721	EI1 Find information	0.821	0.603	0.521	0.869	0.859
	EI2 Receive e-mail	0.814	0.620			
	EI3 Receive results	0.800	0.498			
	EO1 Book appointment	0.795	0.510			
	EO2 Online channels	0.791	0.522			
	EO3 Check effects	0.802	0.514			
	EB1 Billing process	0.812	0.497			
	EB2 Pay bill	0.795	0.488			
	EB3 Receive bill	0.782	0.589			
	EP1 Payment options	0.811	0.523			
	EP2 Payment time	0.810	0.524			
	EP3 Payment methods	0.798	0.610			
	EC1 Customized advices	0.816	0.650			
	EC2 Hospital facilities	0.809	0.556			
	EC3 Staff interactions	0.850	0.543			
PE Mean = 4.09 SD = 0.688 Discriminant Validity Assessment = 0.785	PE1 Online communication	0.822	0.601	0.583	0.839	0.822
	PE2 Adequate of information	0.801	0.622			
	PE3 Online appointment	0.789	0.603			
	PE4 Website navigation	0.781	0.505			
	PE5 Online instructions	0.802	0.516			
PL Mean = 4.01 SD = 0.683 Discriminant Validity Assessment = 0.744	PL1 Flexible appointment	0.810	0.517	0.564	0.851	0.844
	PL2 Quality of consultations	0.792	0.514			
	PL3 Billing system	0.802	0.602			
	PL4 Payment system	0.809	0.501			
	PL5 Information system	0.801	0.506			

FL = Factor Loading, FLS = Factor Loading Squared, AVE = Average Variance Extracted, CR = Composite Reliability, CSS = Cyberspace supplementary health services, EI = E-Information, EO = E-Order Taking, EC = E-Consultation, EB = E-Billing, and EP = E-Payment, PL = Patient loyalty, PE = Patient experience.

**Table 3:** Structural Equation Modelling Regression Weights.

	Path		Estimate	S.E.	C.R.	*p*	Effect	R
CSS	→	PL	0.282	0.038	24.144	***	0.282	0.282 **
CSS	→	PE	0.591	0.039	22.514	***	0.591	0.591 **
PE	→	PL	0.431	0.034	23.284	***	0.431	0.431 **

S.E. = Standard errors of the regression weights, C.R. = Critical Ratio, *p* = *p*-value (** < 0.01, *** < 0.001). CSS = Cyberspace supplementary health services, PL = Patient loyalty, PE = Patient experience.

## Data Availability

The data presented in this study are available on reasonable request from the corresponding author. The data are not publicly available due to ethical and privacy restrictions associated with human participant research and the conditions of approval granted by the Research Ethics Committee of King Abdulaziz University Hospital.

## Supplementary Materials

The following supporting information can be downloaded at: https://www.mdpi.com/article/10.3390/healthcare14162512/s1, File S1: pilot results. File S2: questionnaire items. File S3: discriminant validity assessment results.
