# Supplementary material for: Cyberspace Supplementary Health Services and Patient Loyalty: The Role of Patient Experience in Saudi Arabia
Source: Healthcare (Basel). 2026 Aug 12;14(16):2512. doi: 10.3390/healthcare14162512 (PMC13512494; doi:10.3390/healthcare14162512)
Supplement: Supplementary file 1 [file healthcare-14-02512-s001.zip › healthcare-4419471-supplementary.pdf]

File S1

Expert content validation

The preliminary questionnaire was reviewed by three experts with experience in digital health services, healthcare quality, and health services research. The experts independently evaluated each questionnaire item for relevance, clarity, wording, and cultural appropriateness using a four-point rating scale.

Table S1. Characteristics of expert reviewers

Expert	Area of expertise	Academic qualification	Years of experience
Expert 1	Digital Health	PhD	15
Expert 2	Healthcare Quality	PhD	18
Expert 3	Health Services Research	PhD	12

Table S2. Item-level and scale-level content validity indices of the study questionnaire based on the independent evaluation of three experts with experience in digital healthcare, healthcare quality, and health services research. Experts reviewed each questionnaire item for relevance, clarity, wording, simplicity, and cultural appropriateness using a four-point rating scale (1 = not relevant, 2 = somewhat relevant, 3 = quite relevant, and 4 = highly relevant). The Item-Level Content Validity Index (I-CVI) was calculated as the proportion of experts assigning a relevance rating of either 3 or 4 to each item. The Scale-Level Content Validity Index (S-CVI/Ave) was calculated as the average I-CVI across all items within each construct. Values approaching 1.00 indicate excellent expert agreement regarding the content validity of the questionnaire.

Construct	Number of Items	Mean I-CVI	S-CVI/Ave
E-Information	5	1.00	1.00
E-Order Taking	4	0.92	0.97
E-Consultation	4	1.00	1.00
E-Billing	3	1.00	1.00
E-Payment	3	0.94	0.98
Patient Experience	5	0.96	0.98
Patient Loyalty	4	1.00	1.00

Table S3. Summary of expert recommendations

Issue identified	Recommendation	Action taken
Technical wording	Simplify terminology	Revised wording
Long questions	Reduce sentence length	Items shortened
Redundant phrases	Remove repetitive wording	Text streamlined
Cultural appropriateness	Adapt wording for local healthcare context	Terminology modified
Questionnaire layout	Improve formatting	Layout revised

Table S4. Characteristics of pilot participants (n = 15)

Characteristic	n (%)
Male	8 (53.3)
Female	7 (46.7)
Age (years), mean ± SD	36.8 ± 10.4

Bachelor's degree or higher	11 (73.3)
Previous experience using digital hospital services	15 (100)

Table S5. Summary of participant feedback and questionnaire revisions following pilot testing

**Feedback received**	**Number of participants (n = 15)**	**Action taken**
Some questions were too long and could be simplified.	5	Long sentences were shortened while preserving their meaning.
Minor grammatical or wording issues were identified.	4	Grammar and wording were revised throughout the questionnaire.
Some medical or technical terms required clarification.	3	Simpler and more familiar terminology was adopted.
The questionnaire layout could be improved.	2	Formatting, spacing, and section headings were improved.
No additional comments; questionnaire was easy to complete.	8	No further revisions required.

File S2

Cyberspace supplementary health services and patient loyalty: The role of patient experience in Saudi Arabia

Call for Participation

Dear Adult Patients,

You are invited to participate in a research survey exploring the roles of cyberspace supplementary health services, patient experience, and patient loyalty among adults in Saudi Arabia.

Your participation will help improve the quality of digital health services and patient-centered care in healthcare organizations.

🕒 **Time to complete:** Approximately **10 minutes**

👥 **Target participants: Adults (18 years and above)** who have used digital health services (e.g., online appointments, e-billing, e-payment, or online communication)

🧮 **Method:** Online questionnaire

🔒 **Participation:** Voluntary and anonymous

For any questions, please contact:
nalsubahi@kau.edu.sa

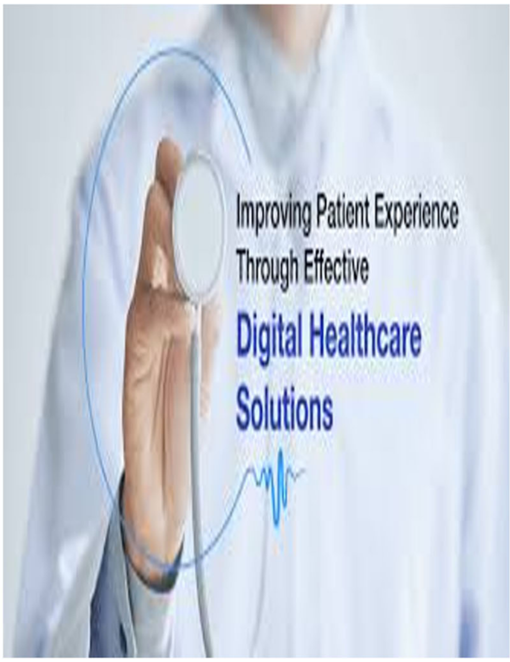

A. ELECTRONIC CONSENT FORM

I confirm that I have read the information sheet dated 01/10/2025 version 1 for the above research project.
I confirm that I have understood the information sheet dated 01/10/2025 version 1 for the above research project and that I have had the opportunity to ask questions if I did not understand any of the questions by contacting the main author.
I understand that my participation is voluntary, and I am free to withdraw at any time without giving a reason and without any adverse consequences (e.g. to medical care or legal rights, if relevant).
I understand that data collected during the research project may be looked at by the research team, where it is relevant to my taking part in the research project. I give permission for these individuals to have access to my answers.
I consent to the processing of my personal information, such as my income, for the purposes explained to me. I understand that such information will be held in accordance with all applicable data protection legislation and in strict confidence, unless law or professional obligation requires disclosure.
I understand that after the research project, anonymized data may be published and made publicly available via a data repository and may be used for purposes not related to this research project, such as academic research papers and presentations. I understand it will not be possible to identify me from the data seen and used by other researchers, for ethically approved research projects, on the understanding that confidentiality will be maintained.
I understand how the findings and results of the research project will be written up and published.

I agree to participate ……   I do not agree to participate …….

Thank you so much for participating in this research!

Part One: Demographic information about participants:

Gender:
□ Female
□ Male

Educational Level:
□ Diploma or less
□ Bachelor Degree
□ High Degrees

Age:
□ 20 years or Less
□ 21-30
□ 31-40
□41-50
□ 51and more

Technology knowledge Level:
□ High knowledge in technology
□ Medium knowledge in technology
□ Low knowledge in technology

Part Two: Research Dimensions related Questions

	**E-Information**
Q1	I can easily find information regarding (e.g. healthcare providers, prices…) on the hospital webpage<br>□ Strongly Agree □ Agree □ Neutral □ Disagree<br>□ Strongly Disagree
Q2	I receive email notice from the hospital informing me about any new promotion offers (e.g. Lab tests).<br>□ Strongly Agree □ Agree □ Neutral □ Disagree<br>□ Strongly Disagree
Q3	I can easily receive the results of diagnostic tests.<br>□ Strongly Agree □ Agree □ Neutral □ Disagree<br>□ Strongly Disagree
	**E-Order Taking (E-Appointment)**
Q1	I can easily book my appointment online.<br>□ Strongly Agree □ Agree □ Neutral □ Disagree<br>□ Strongly Disagree
Q2	I can easily use online channels to ask my doctor questions.<br>□ Strongly Agree □ Agree □ Neutral □ Disagree<br>□ Strongly Disagree
Q3	I can easily check the effects and side-effects of a medicine.<br>□ Strongly Agree □ Agree □ Neutral □ Disagree<br>□ Strongly Disagree
	**E-Billing**

Q1	I can easily deal with my billing process through the webpage.<br>□ Strongly Agree □ Agree □ Neutral □ Disagree<br>□ Strongly Disagree
Q2	I can easily pay the bill online.<br>□ Strongly Agree □ Agree □ Neutral □ Disagree<br>□ Strongly Disagree
Q3	I receive a detailed bill on my e-mail.<br>□ Strongly Agree □ Agree □ Neutral □ Disagree<br>□ Strongly Disagree
**E-Payment**	
Q1	I can easily find a variety of options when I want to pay (Direct debit/ credit card, Cliq, by email, and PayPal).<br>□ Strongly Agree □ Agree □ Neutral □ Disagree<br>□ Strongly Disagree
Q2	E-payment makes fees payment faster than before.<br>□ Strongly Agree □ Agree □ Neutral □ Disagree<br>□ Strongly Disagree
Q3	I recive paying points or cashback on my e-payment.<br>□ Strongly Agree □ Agree □ Neutral □ Disagree<br>□ Strongly Disagree
**E-Consultation**	
Q1	I receive customized medical advice via email messages.<br>□ Strongly Agree □ Agree □ Neutral □ Disagree<br>□ Strongly Disagree
Q2	I receive emails about use of the hospital facilities.<br>□ Strongly Agree □ Agree □ Neutral □ Disagree<br>□ Strongly Disagree

Q3	My interaction with health care providers is clear and understandable.<br>□ Much less than competitors □ Less than competitors □ The same as competitors □ More than competitors □ Much more than competitors
**Patient Experience**	
Q1	How would you rate the clarity of the communication you received from your healthcare provider?<br>□ Strongly Agree □ Agree □ Neutral □ Disagree<br>□ Strongly Disagree
Q2	Were you provided with adequate information about your medications, including their purpose and potential side effects?<br>□ Strongly Agree □ Agree □ Neutral □ Disagree<br>□ Strongly Disagree
Q3	Were you provided with clear online instructions on how to prepare for your appointment (e.g., fasting requirements, medication restrictions)?<br>□ Strongly Agree □ Agree □ Neutral □ Disagree<br>□ Strongly Disagree
Q4	Was the website comfortable and easyto navigate?<br>□ Strongly Agree □ Agree □ Neutral □ Disagree<br>□ Strongly Disagree
Q5	Were you provided with clear online instructions on how to manage your condition at home?

	□ Strongly Agree □ Agree □ Neutral □ Disagree<br>□ Strongly Disagree
**Patient Loyalty**	
Q1	I am satisfied with the flexible appointment system in the hospital departments.<br>□ Strongly Agree □ Agree □ Neutral □ Disagree<br>□ Strongly Disagree
Q2	I am satisfied with the consultations provided by the medical staff.<br>□ Strongly Agree □ Agree □ Neutral □ Disagree<br>□ Strongly Disagree
Q3	I am satisfied with the electronic billing system in the hospital departments.<br>□ Strongly Agree □ Agree □ Neutral □ Disagree<br>□ Strongly Disagree
Q4	I am satisfied with the electronic payment system in place.<br>□ Strongly Agree □ Agree □ Neutral □ Disagree<br>□ Strongly Disagree
Q5	I am satisfied with the information system and its availability in the hospital.<br>□ Strongly Agree □ Agree □ Neutral □ Disagree<br>□ Strongly Disagree

The research questionnaire constructs and measurement items were adapted from the relevant literature and validated scales reported in the following references, ensuring their alignment with the objectives

1- E-information: Wirtz and Lovelock, 2016; Ahmad et al., 2022; Hashem et al., 2018; Sun et al., 2022; Zhang et al., 2020.

2- Taking E-Order (E-Appointment): Wirtz and Lovelock, 2016; Ahmad et al., 2022; Hashem et al., 2018; Sun et al., 2022; Zhang et al., 2020; Cao et al., 2018; Mahfouz et al., 2023.

3- E-Billing: Wirtz and Lovelock, 2016; Ahmad et al., 2022; Hashem et al., 2018; Smith et al. (2017) ; Johnson et al. (2018); Brown et al. (2019); Kim et al. (2021); Gupta et al. (2022).

4- E-Payment: Wirtz and Lovelock, 2016; Ahmad et al., 2022; Hashem et al., 2018; Chen et al., 2021; Gupta et al., 2020; Lee & Park, (2021; (Patel & Sharma, 2024).

5- E-Consultation: Wirtz and Lovelock, 2016; Ahmad et al., 2022; Hashem et al., 2018; Barnett et al. (2018); Liddy et al. (2016); Keely et al. (2019)

6- Patient Experience: (Bleich et al., 2017). Gerteis et al., 2015; (Merriel et al., 2019; McCleery et al., 2021)

7-Patient Loyalty: Astuti, H. J., & Nagase, K. (2016). Kim, J., et al. (2019).

File S3

The research model consists of seven latent constructs, the analyses include:

Table S6. Construct correlation matrix. E-Information (EI), E-Order Taking (EO), E-Consultation (EC), E-Billing (EB), E-Payment (EP), Patient Experience (PE), Patient Loyalty (PL). All inter-construct correlations are below 0.85, indicating that multicollinearity is unlikely and providing preliminary evidence of discriminant validity.

Construct	EI	EO	EC	EB	EP	PE	PL
EI	1.000						
EO	0.624	1.000					
EC	0.579	0.631	1.000				
EB	0.545	0.563	0.488	1.000			
EP	0.492	0.527	0.486	0.614	1.000		
PE	0.652	0.619	0.701	0.574	0.618	1.000	
PL	0.467	0.541	0.565	0.448	0.524	0.718	1.000

Table S7. Fornell–Larcker criterion. The square root of the AVE for each construct exceeds its correlations with all other constructs, satisfying the Fornell–Larcker criterion and confirming discriminant validity. E-Information (EI), E-Order Taking (EO), E-Consultation (EC), E-Billing (EB), E-Payment (EP), Patient Experience (PE), Patient Loyalty (PL).

Construct	EI	EO	EC	EB	EP	PE	PL
EI	**0.861***						
EO	0.624	**0.882***					
EC	0.579	0.631	**0.913***				
EB	0.545	0.563	0.488	**0.889***			
EP	0.492	0.527	0.486	0.614	**0.892***		
PE	0.652	0.619	0.701	0.574	0.618	**0.925***	
PL	0.467	0.541	0.565	0.448	0.524	0.718	**0.911***

Diagonal values (*) represent the square root of the Average Variance Extracted (AVE).

Table S8. The Heterotrait-Monotrait **(**HTMT) ratios results. E-Information (EI), E-Order Taking (EO), E-Consultation (EC), E-Billing (EB), E-Payment (EP), Patient Experience (PE), Patient Loyalty (PL).

**Constructs**	**HTMT**
EI – EO	0.794
EI – EC	0.668
EI – EB	0.589
EI – EP	0.566
EI – PE	0.743
EI – PL	0.575
EO – EC	0.734
EO – EB	0.620
EO – EP	0.581
EO – PE	0.762
EO – PL	0.584
EC – EB	0.564
EC – EP	0.536
EC – PE	0.787
EC – PL	0.618
EB – EP	0.716
EB – PE	0.672
EB – PL	0.543
EP – PE	0.691
EP – PL	0.561
PE – PL	0.825

All HTMT values are below the conservative threshold of 0.85 (and well below the liberal threshold of 0.90), indicating satisfactory discriminant validity among all constructs, including the conceptually related constructs of Patient Experience and Patient Loyalty.

Discriminant validity was assessed using three complementary approaches: the construct correlation matrix, the Fornell–Larcker criterion, and the Heterotrait–Monotrait (HTMT) ratio. All inter-construct correlations were below the recommended threshold of 0.85. Furthermore, the square root of the AVE for each construct exceeded its correlations with all other constructs,

thereby satisfying the Fornell–Larcker criterion. Finally, all HTMT values were below the recommended cutoff value of 0.85, with the highest HTMT value observed between Patient Experience and Patient Loyalty (HTMT = 0.825). Collectively, these findings provide strong evidence that the constructs exhibit adequate discriminant validity despite the conceptual relatedness of Patient Experience and Patient Loyalty.
